# 1′-(1,3-Diphenyl-1*H*-pyrazol-4-yl)-2′,3′,5′,6′,7′,7a’-hexa­hydro-1′*H*-di­spiro­[ace­naphthyl­ene-1,3′-pyrrolizine-2′,3′′-chromane]-2,4′′(1*H*)-dione

**DOI:** 10.1107/S1600536813009562

**Published:** 2013-04-13

**Authors:** G. Jagadeesan, D. Kathirvelan, J. Haribabu, B. S. R. Reddy, K. Sethusankar

**Affiliations:** a Department of Physics, Meenakshi College of Engineering, West K.K. Nagar, Chennai 600 078, India; b Industrial Chemistry Lab, Central Leather Research Institute, Adyar, Chennai 600 020, India; c Department of Physics, RKM Vivekananda College (Autonomous), Chennai 600 004, India

## Abstract

In the title compound, C_41_H_31_N_3_O_3_, the pyrazole and pyrrolidine rings adopt twisted conformations. The mean plane of the pyrazole ring forms dihedral angles of 9.11 (12) and 39.65 (11)° with the phenyl rings. The O atoms deviate from the mean planes of the chromene and ace­naphthyl­ene ring systems by 0.194 (15) and 0.079 (15) Å, respectively. In the crystal, molecules are linked *via* pairs of C—H⋯O inter­actions,forming inversion dimers with an *R*_2_^2^(12) ring motif.

## Related literature

For the biological activity of pyrazole derivatives, see: Mahajan *et al.* (1991 ▶); Baraldi *et al.* (1998 ▶); Katayama & Oshiyama (1997 ▶); Chen & Li (1998 ▶). For a related structure, see: Jagadeesan *et al.* (2013 ▶). For puckering parameters, see: Cremer & Pople (1975 ▶). For graph-set notation, see: Bernstein *et al.* (1995 ▶).
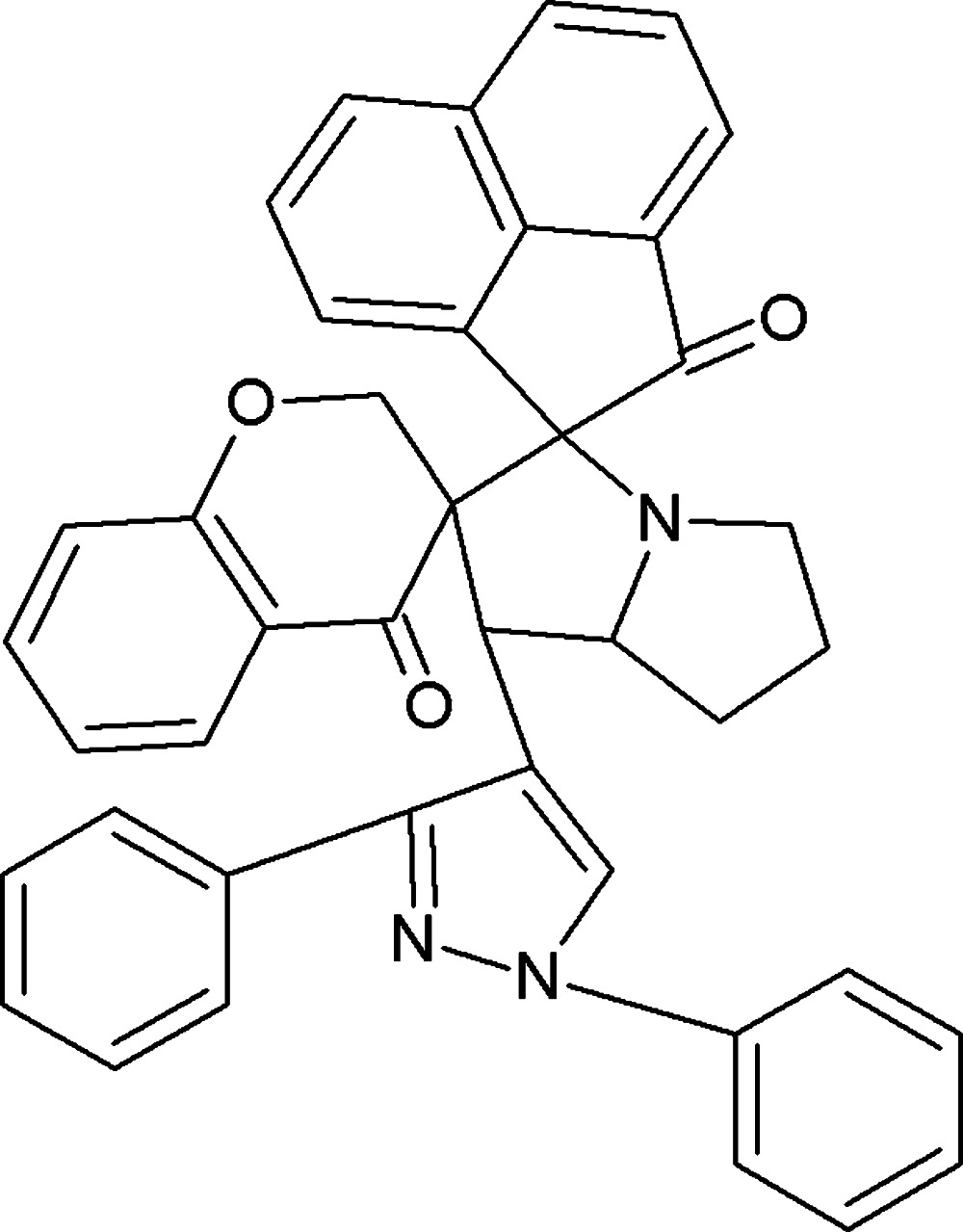


## Experimental

### 

#### Crystal data

C_41_H_31_N_3_O_3_
*M* *_r_* = 613.69
Triclinic, 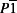

*a* = 10.0183 (4) Å
*b* = 12.7374 (5) Å
*c* = 13.2489 (5) Å
α = 114.365 (2)°
β = 96.960 (2)°
γ = 92.281 (2)°
*V* = 1521.17 (11) Å^3^
*Z* = 2
Mo *K*α radiation
μ = 0.09 mm^−1^
*T* = 295 K
0.30 × 0.25 × 0.20 mm

#### Data collection

Bruker Kappa APEXII CCD diffractometer
Absorption correction: multi-scan (*SADABS*; Bruker, 2008 ▶) *T*_min_ = 0.975, *T*_max_ = 0.983
32812 measured reflections
9207 independent reflections
5094 reflections with *I* > 2σ(*I*)
*R*_int_ = 0.036

#### Refinement

*R*[*F* ^2^ > 2σ(*F* ^2^)] = 0.055
*wR*(*F* ^2^) = 0.166
*S* = 1.01
9207 reflections
424 parameters
H-atom parameters constrained
Δρ_max_ = 0.46 e Å^−3^
Δρ_min_ = −0.32 e Å^−3^

### 

Data collection: *APEX2* (Bruker, 2008 ▶); cell refinement: *SAINT* (Bruker, 2008 ▶); data reduction: *SAINT*; program(s) used to solve structure: *SHELXS97* (Sheldrick, 2008 ▶); program(s) used to refine structure: *SHELXL97* (Sheldrick, 2008 ▶); molecular graphics: *ORTEP-3 for Windows* (Farrugia, 2012 ▶); software used to prepare material for publication: *SHELXL97* and *PLATON* (Spek, 2009 ▶).

## Supplementary Material

Crystal structure: contains datablock(s) global, I. DOI: 10.1107/S1600536813009562/rk2396sup1.cif

Structure factors: contains datablock(s) I. DOI: 10.1107/S1600536813009562/rk2396Isup2.hkl

Additional supplementary materials:  crystallographic information; 3D view; checkCIF report

## Figures and Tables

**Table 1:** Hydrogen-bond geometry (Å, °)

*D*—H⋯*A*	*D*—H	H⋯*A*	*D*⋯*A*	*D*—H⋯*A*
C22—H22⋯O3^i^	0.98	2.47	3.273 (2)	138

Symmetry code: (i) 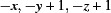.

**Fig. 1.**
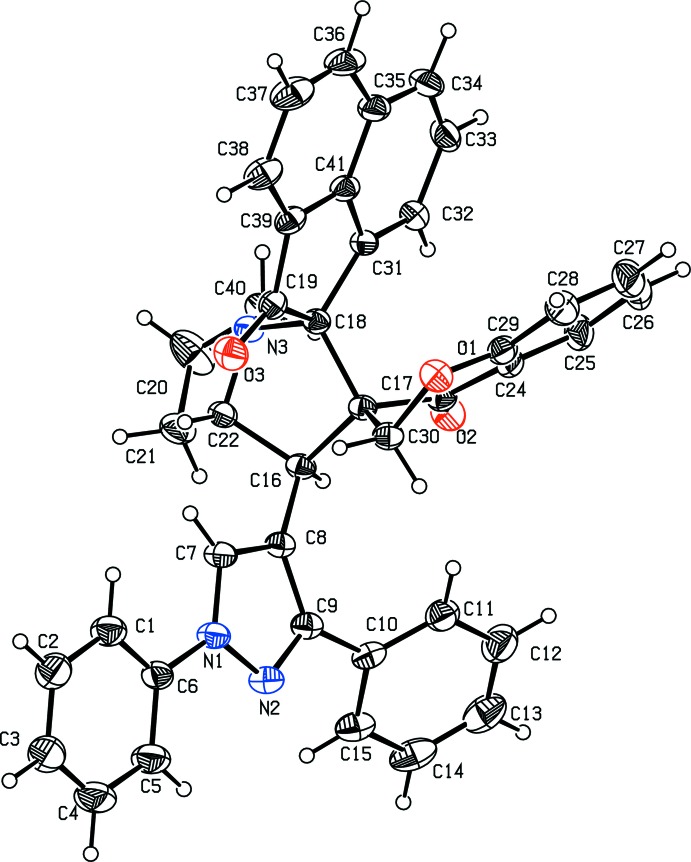
The molecular structure of the title compound with the atom numbering scheme. Displacement ellipsoids are drawn at 30% probability level. H atoms are presented as a small spheres of arbitrary radius.

**Fig. 2.**
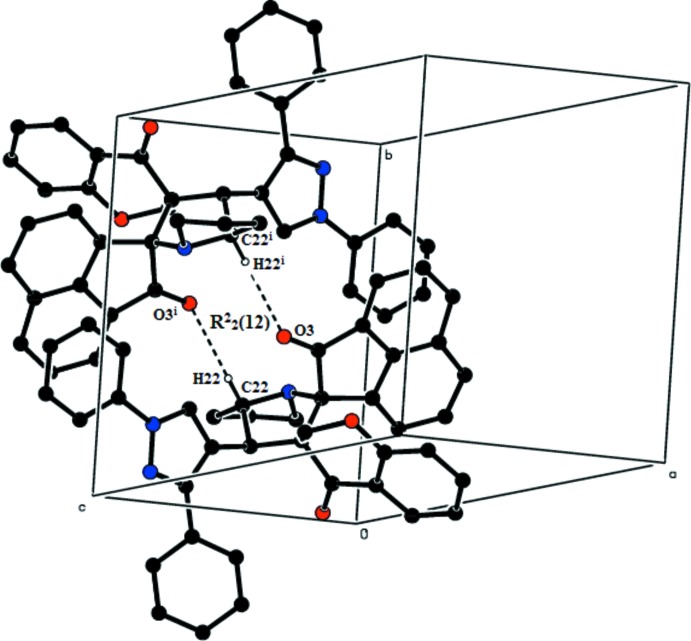
The crystal packing of the title compound viewed down b axis, showing the hydrogen bonds resulting in R22(12) graph–set ring motif. H atoms not involved in hydrogen bonds have been excluded for clarity.

## supplementary crystallographic information

### Comment

Pyrazole derivatives in general are well known nitrogen containing heterocyclic compounds and have been the subject of enormous research due to their importance in various applications and their widespread potential biological and pharmacological activities such as antimicrobial (Mahajan *et al.*, 1991), antiviral (Baraldi *et al.*, 1998), antitumor (Katayama & Oshiyama, 1997) and antifungal activities (Chen & Li, 1998).

The molecular structure of the title compound C_41_H_31_N_3_O_3_, is shown in Fig. 1. The mean planes of the phenyl rings (C1–C6) and (C10–C15) form a dihedral angle of 31.31 (12)° between them. The mean plane of the pyrazole ring (C7/C8/C9/N1/N2) forms dihedral angles of 9.11 (12)° and 39.65 (11)° with the mean planes of the two phenyl rings (C1–C6) and (C10–C15), respectively. The mean plane of the pyrrolizine ring (C16–C22/N3) forms dihedral angles of 63.95 (9)° and 74.46 (8)° with the mean planes of the chromene ring (C17/C23–C30/O1) and the acenaphthylene ring (C18/C31–C41), respectively. The oxygen atoms O2 and O3 deviate from the least square planes of the chromene ring (C17/C23–C30/O1) and acenaphthylene ring (C18/C31–C41) by -0.194 (15)Å and -0.079 (15)Å, respectively. The title compound exhibits structural similarities with an already reported related structure (Jagadeesan *et al.*, 2013).

The sum of angles around the N_3_ atom (335.6°) indicates *sp*^3^ hybridization. The pyrrolidine ring (C16/C17/C18/C22/N3) adopts a *twisted* conformation on C22 and N3, with puckering parameters (Cremer & Pople, 1975) of q_2_ = 0.322 (18)Å and φ_2_ = 170.9 (3)°. Also, the atoms C22 and N3 deviate from the mean planes of the remaining ring atoms by 0.182 (2)Å and -0.201 (16)Å, respectively. The other pyrrolidine ring (C19–C22/N3) also adopts a *twisted* conformation on C19 and N3 with puckering parameters of q_2_ = 0.382 (2)Å and φ_2_ = 205.1 (4)°. Also, the atoms C19 and N3 deviate from the mean planes of the remaining ring atoms by 0.237 (2)Å and -0.219 (18)Å, respectively.

The crystal packing is stabilized by C22—H22···O3^i^ intermolecular hydrogen bond interaction, which generates *R*^2^_2_(12) graphset ring motif (Bernstein, *et al.*, 1995). The symmetry codes: (i) -*x*, *y*-1, *z*-1 (Table 1). The packing view of the title compound is shown in Fig. 2.

### Experimental

A mixture of acenaphthenequinone (1.05 mmol), *L*–proline (1.1 mmol) and dipolarophile (1.0 mmol) in ethanol was refluxed for 5 hrs and cooled to room temperature. The solid formed in the reaction mixture was poured into a beaker containing crushed ice and it was filtered, dried, and recrystallized from ethanol to obtain the pure product in good yield (89%) without involving column chromatography.

### Refinement

Hydrogen atoms were placed in calculated positions with C—H = 0.93Å to 0.98Å and refined in the riding model with fixed isotropic displacement parameters: *U*_iso_(H) = 1.5*U*_eq_(C) for methyl group and *U*_iso_(H) = 1.2*U*_eq_(C) for other groups.

### Crystal data

**Table:**

C_41_H_31_N_3_O_3_	*Z* = 2
*M**_r_* = 613.69	*F*(000) = 644
Triclinic, *P*1	*D*_x_ = 1.340 Mg m^−^^3^
Hall symbol: -P 1	Mo *K*α radiation, λ = 0.71073 Å
*a* = 10.0183 (4) Å	Cell parameters from 9207 reflections
*b* = 12.7374 (5) Å	θ = 2.1–30.9°
*c* = 13.2489 (5) Å	µ = 0.09 mm^−^^1^
α = 114.365 (2)°	*T* = 295 K
β = 96.960 (2)°	Block, colourless
γ = 92.281 (2)°	0.30 × 0.25 × 0.20 mm
*V* = 1521.17 (11) Å^3^	

### Data collection

**Table:**

Bruker Kappa APEXII CCD diffractometer	9207 independent reflections
Radiation source: fine–focus sealed tube	5094 reflections with *I* > 2σ(*I*)
Graphite monochromator	*R*_int_ = 0.036
ω scans	θ_max_ = 30.9°, θ_min_ = 2.1°
Absorption correction: multi-scan (*SADABS*; Bruker, 2008)	*h* = −14→14
*T*_min_ = 0.975, *T*_max_ = 0.983	*k* = −18→17
32812 measured reflections	*l* = −19→18

### Refinement

**Table:**

Refinement on *F*^2^	Primary atom site location: structure-invariant direct methods
Least-squares matrix: full	Secondary atom site location: difference Fourier map
*R*[*F*^2^ > 2σ(*F*^2^)] = 0.055	Hydrogen site location: inferred from neighbouring sites
*wR*(*F*^2^) = 0.166	H-atom parameters constrained
*S* = 1.01	*w* = 1/[σ^2^(*F*_o_^2^) + (0.0652*P*)^2^ + 0.4938*P*] where *P* = (*F*_o_^2^ + 2*F*_c_^2^)/3
9207 reflections	(Δ/σ)_max_ < 0.001
424 parameters	Δρ_max_ = 0.46 e Å^−^^3^
0 restraints	Δρ_min_ = −0.32 e Å^−^^3^

### Special details

**Table:**

Geometry. All s.u.'s (except the s.u. in the dihedral angle between two l.s. planes) are estimated using the full covariance matrix. The cell s.u.'s are taken into account individually in the estimation of s.u.'s in distances, angles and torsion angles; correlations between s.u.'s in cell parameters are only used when they are defined by crystal symmetry. An approximate (isotropic) treatment of cell s.u.'s is used for estimating s.u.'s involving l.s. planes.
Refinement. Refinement of *F*^2^ against ALL reflections. The weighted *R*–factor *wR* and goodness of fit *S* are based on *F*^2^, conventional *R*–factors *R* are based on *F*, with *F* set to zero for negative *F*^2^. The threshold expression of *F*^2^ > σ(*F*^2^) is used only for calculating *R*–factors(gt) *etc*. and is not relevant to the choice of reflections for refinement. *R*–factors based on *F*^2^ are statistically about twice as large as those based on *F*, and *R*–factors based on ALL data will be even larger.

### Fractional atomic coordinates and isotropic or equivalent isotropic displacement parameters (Å^2^)

**Table:**

	*x*	*y*	*z*	*U*_iso_*/*U*_eq_	
C1	−0.1153 (2)	0.41978 (18)	0.78887 (17)	0.0533 (5)	
H1	−0.0616	0.4537	0.7557	0.064*	
C2	−0.1402 (3)	0.4823 (2)	0.89637 (18)	0.0632 (6)	
H2	−0.1034	0.5590	0.9355	0.076*	
C3	−0.2181 (3)	0.4336 (2)	0.94646 (19)	0.0736 (7)	
H3	−0.2341	0.4764	1.0193	0.088*	
C4	−0.2722 (3)	0.3214 (2)	0.8885 (2)	0.0794 (8)	
H4	−0.3255	0.2880	0.9223	0.095*	
C5	−0.2492 (3)	0.2568 (2)	0.78067 (18)	0.0643 (6)	
H5	−0.2865	0.1803	0.7420	0.077*	
C6	−0.1705 (2)	0.30644 (16)	0.73071 (15)	0.0437 (4)	
C7	−0.09357 (19)	0.27729 (16)	0.54960 (15)	0.0425 (4)	
H7	−0.0573	0.3524	0.5680	0.051*	
C8	−0.10027 (17)	0.18514 (15)	0.44788 (14)	0.0372 (4)	
C9	−0.16394 (18)	0.09198 (15)	0.46325 (15)	0.0403 (4)	
C10	−0.20193 (19)	−0.02968 (16)	0.38356 (15)	0.0428 (4)	
C11	−0.1185 (2)	−0.09176 (17)	0.30846 (17)	0.0525 (5)	
H11	−0.0359	−0.0560	0.3081	0.063*	
C12	−0.1555 (3)	−0.20514 (19)	0.2345 (2)	0.0686 (6)	
H12	−0.0981	−0.2455	0.1847	0.082*	
C13	−0.2772 (3)	−0.2589 (2)	0.2341 (2)	0.0758 (7)	
H13	−0.3030	−0.3354	0.1834	0.091*	
C14	−0.3603 (3)	−0.1999 (2)	0.3083 (2)	0.0711 (7)	
H14	−0.4423	−0.2368	0.3084	0.085*	
C15	−0.3240 (2)	−0.08581 (18)	0.38343 (18)	0.0553 (5)	
H15	−0.3811	−0.0466	0.4339	0.066*	
C16	−0.06665 (17)	0.18964 (14)	0.34183 (13)	0.0349 (4)	
H16	−0.1214	0.1255	0.2785	0.042*	
C17	0.08327 (16)	0.18237 (13)	0.32134 (13)	0.0320 (3)	
C18	0.11508 (16)	0.29091 (13)	0.29340 (13)	0.0323 (3)	
C19	−0.0864 (2)	0.2779 (2)	0.15276 (16)	0.0521 (5)	
H19A	−0.0823	0.1948	0.1179	0.063*	
H19B	−0.0498	0.3122	0.1075	0.063*	
C20	−0.2280 (2)	0.3075 (3)	0.1699 (2)	0.0799 (8)	
H20A	−0.2928	0.2529	0.1082	0.096*	
H20B	−0.2389	0.3849	0.1752	0.096*	
C21	−0.2478 (2)	0.3008 (2)	0.27781 (18)	0.0551 (5)	
H21A	−0.3012	0.2294	0.2632	0.066*	
H21B	−0.2932	0.3657	0.3235	0.066*	
C22	−0.10583 (17)	0.30447 (15)	0.33630 (14)	0.0374 (4)	
H22	−0.0921	0.3673	0.4120	0.045*	
C23	0.10449 (18)	0.07340 (14)	0.22056 (14)	0.0366 (4)	
C24	0.24610 (18)	0.04615 (14)	0.21767 (15)	0.0396 (4)	
C25	0.2856 (2)	−0.03313 (16)	0.11913 (18)	0.0526 (5)	
H25	0.2201	−0.0770	0.0582	0.063*	
C26	0.4192 (2)	−0.04729 (19)	0.1109 (2)	0.0639 (6)	
H26	0.4444	−0.0996	0.0445	0.077*	
C27	0.5164 (2)	0.0169 (2)	0.2020 (2)	0.0649 (6)	
H27	0.6071	0.0071	0.1965	0.078*	
C28	0.4813 (2)	0.09427 (18)	0.29976 (19)	0.0547 (5)	
H28	0.5475	0.1365	0.3607	0.066*	
C29	0.34599 (19)	0.10968 (15)	0.30785 (16)	0.0416 (4)	
C30	0.17775 (17)	0.18192 (15)	0.42002 (14)	0.0383 (4)	
H30A	0.1633	0.2464	0.4882	0.046*	
H30B	0.1568	0.1109	0.4280	0.046*	
C31	0.20696 (17)	0.28246 (14)	0.20732 (14)	0.0349 (4)	
C32	0.2028 (2)	0.21023 (16)	0.09670 (15)	0.0429 (4)	
H32	0.1360	0.1485	0.0607	0.051*	
C33	0.3023 (2)	0.23111 (19)	0.03788 (17)	0.0535 (5)	
H33	0.3003	0.1810	−0.0370	0.064*	
C34	0.4005 (2)	0.3215 (2)	0.0865 (2)	0.0577 (6)	
H34	0.4637	0.3320	0.0447	0.069*	
C35	0.40668 (19)	0.39868 (17)	0.19933 (18)	0.0481 (5)	
C36	0.4972 (2)	0.4997 (2)	0.2628 (2)	0.0623 (6)	
H36	0.5643	0.5191	0.2290	0.075*	
C37	0.4887 (2)	0.5686 (2)	0.3713 (2)	0.0659 (6)	
H37	0.5504	0.6338	0.4099	0.079*	
C38	0.3901 (2)	0.54461 (17)	0.42660 (18)	0.0534 (5)	
H38	0.3850	0.5931	0.5008	0.064*	
C39	0.30067 (18)	0.44744 (14)	0.36843 (15)	0.0393 (4)	
C40	0.18767 (18)	0.39757 (14)	0.40065 (14)	0.0373 (4)	
C41	0.30899 (17)	0.37581 (15)	0.25702 (15)	0.0379 (4)	
N1	−0.14891 (16)	0.24040 (13)	0.61905 (12)	0.0436 (4)	
N2	−0.19339 (16)	0.12621 (14)	0.56655 (13)	0.0459 (4)	
N3	−0.01547 (14)	0.32956 (12)	0.26812 (11)	0.0361 (3)	
O1	0.31681 (12)	0.19063 (11)	0.40564 (10)	0.0439 (3)	
O2	0.01396 (13)	0.01606 (11)	0.14478 (11)	0.0511 (3)	
O3	0.15203 (14)	0.43234 (11)	0.49191 (10)	0.0507 (3)	

### Atomic displacement parameters (Å^2^)

**Table:**

	*U*^11^	*U*^22^	*U*^33^	*U*^12^	*U*^13^	*U*^23^
C1	0.0621 (14)	0.0563 (12)	0.0474 (11)	0.0030 (10)	0.0128 (10)	0.0266 (10)
C2	0.0849 (17)	0.0556 (12)	0.0506 (12)	0.0100 (12)	0.0129 (11)	0.0230 (10)
C3	0.114 (2)	0.0691 (15)	0.0485 (13)	0.0231 (15)	0.0335 (13)	0.0283 (12)
C4	0.116 (2)	0.0733 (16)	0.0641 (15)	0.0083 (15)	0.0459 (15)	0.0349 (13)
C5	0.0867 (18)	0.0577 (13)	0.0539 (13)	−0.0010 (12)	0.0277 (12)	0.0251 (11)
C6	0.0486 (11)	0.0510 (10)	0.0398 (10)	0.0086 (9)	0.0115 (8)	0.0256 (8)
C7	0.0453 (11)	0.0444 (9)	0.0410 (10)	−0.0028 (8)	0.0108 (8)	0.0206 (8)
C8	0.0335 (9)	0.0406 (9)	0.0402 (9)	−0.0006 (7)	0.0071 (7)	0.0199 (8)
C9	0.0357 (10)	0.0462 (10)	0.0434 (10)	−0.0006 (8)	0.0047 (7)	0.0240 (8)
C10	0.0451 (11)	0.0427 (9)	0.0462 (10)	−0.0039 (8)	−0.0006 (8)	0.0271 (8)
C11	0.0531 (12)	0.0473 (11)	0.0598 (12)	0.0014 (9)	0.0079 (10)	0.0257 (10)
C12	0.0844 (18)	0.0481 (12)	0.0704 (15)	0.0061 (12)	0.0121 (13)	0.0221 (11)
C13	0.101 (2)	0.0458 (12)	0.0721 (17)	−0.0132 (13)	−0.0039 (15)	0.0224 (12)
C14	0.0718 (17)	0.0608 (14)	0.0816 (17)	−0.0266 (12)	−0.0100 (13)	0.0397 (13)
C15	0.0515 (13)	0.0581 (12)	0.0623 (13)	−0.0097 (10)	0.0008 (10)	0.0349 (11)
C16	0.0316 (9)	0.0373 (8)	0.0338 (8)	−0.0038 (7)	0.0047 (7)	0.0138 (7)
C17	0.0308 (8)	0.0325 (8)	0.0314 (8)	−0.0023 (6)	0.0039 (6)	0.0130 (6)
C18	0.0326 (9)	0.0311 (8)	0.0313 (8)	−0.0018 (6)	0.0053 (6)	0.0115 (6)
C19	0.0418 (11)	0.0764 (14)	0.0397 (10)	0.0079 (10)	0.0046 (8)	0.0261 (10)
C20	0.0464 (13)	0.139 (2)	0.0582 (14)	0.0239 (15)	0.0082 (11)	0.0436 (16)
C21	0.0356 (10)	0.0747 (14)	0.0698 (14)	0.0089 (10)	0.0132 (9)	0.0431 (12)
C22	0.0356 (9)	0.0424 (9)	0.0384 (9)	0.0043 (7)	0.0108 (7)	0.0196 (7)
C23	0.0365 (9)	0.0325 (8)	0.0380 (9)	−0.0042 (7)	0.0036 (7)	0.0134 (7)
C24	0.0383 (10)	0.0309 (8)	0.0479 (10)	0.0017 (7)	0.0081 (8)	0.0147 (7)
C25	0.0514 (12)	0.0360 (9)	0.0587 (12)	0.0037 (9)	0.0125 (10)	0.0071 (9)
C26	0.0581 (14)	0.0501 (12)	0.0764 (16)	0.0165 (11)	0.0256 (12)	0.0146 (11)
C27	0.0430 (12)	0.0645 (14)	0.0887 (17)	0.0130 (11)	0.0196 (12)	0.0304 (13)
C28	0.0371 (11)	0.0583 (12)	0.0691 (14)	0.0038 (9)	0.0051 (10)	0.0280 (11)
C29	0.0397 (10)	0.0383 (9)	0.0503 (11)	0.0023 (8)	0.0063 (8)	0.0223 (8)
C30	0.0355 (9)	0.0416 (9)	0.0383 (9)	−0.0013 (7)	0.0031 (7)	0.0184 (7)
C31	0.0345 (9)	0.0371 (8)	0.0375 (9)	0.0032 (7)	0.0086 (7)	0.0192 (7)
C32	0.0456 (11)	0.0431 (9)	0.0418 (10)	0.0071 (8)	0.0134 (8)	0.0175 (8)
C33	0.0592 (13)	0.0643 (13)	0.0472 (11)	0.0235 (11)	0.0248 (10)	0.0276 (10)
C34	0.0461 (12)	0.0742 (14)	0.0745 (15)	0.0148 (11)	0.0298 (11)	0.0466 (13)
C35	0.0345 (10)	0.0578 (11)	0.0693 (13)	0.0075 (9)	0.0144 (9)	0.0418 (11)
C36	0.0364 (11)	0.0723 (15)	0.0976 (19)	−0.0077 (10)	0.0085 (11)	0.0565 (15)
C37	0.0494 (13)	0.0618 (14)	0.0882 (18)	−0.0205 (11)	−0.0091 (12)	0.0409 (14)
C38	0.0507 (12)	0.0460 (11)	0.0600 (12)	−0.0131 (9)	−0.0089 (10)	0.0252 (10)
C39	0.0366 (10)	0.0350 (8)	0.0466 (10)	−0.0043 (7)	−0.0011 (7)	0.0200 (8)
C40	0.0390 (10)	0.0317 (8)	0.0383 (9)	0.0005 (7)	0.0030 (7)	0.0128 (7)
C41	0.0291 (9)	0.0408 (9)	0.0508 (10)	0.0014 (7)	0.0051 (7)	0.0265 (8)
N1	0.0486 (9)	0.0462 (8)	0.0400 (8)	−0.0010 (7)	0.0120 (7)	0.0212 (7)
N2	0.0475 (10)	0.0481 (9)	0.0456 (9)	−0.0044 (7)	0.0078 (7)	0.0239 (7)
N3	0.0358 (8)	0.0411 (7)	0.0343 (7)	0.0025 (6)	0.0074 (6)	0.0184 (6)
O1	0.0336 (7)	0.0495 (7)	0.0429 (7)	−0.0019 (5)	−0.0004 (5)	0.0160 (6)
O2	0.0429 (8)	0.0481 (7)	0.0444 (7)	−0.0081 (6)	0.0019 (6)	0.0042 (6)
O3	0.0584 (9)	0.0435 (7)	0.0382 (7)	−0.0038 (6)	0.0090 (6)	0.0057 (6)

### Geometric parameters (Å, º)

**Table:**

C1—C2	1.375 (3)	C20—H20A	0.9700
C1—C6	1.378 (3)	C20—H20B	0.9700
C1—H1	0.9300	C21—C22	1.524 (3)
C2—C3	1.365 (3)	C21—H21A	0.9700
C2—H2	0.9300	C21—H21B	0.9700
C3—C4	1.362 (4)	C22—N3	1.467 (2)
C3—H3	0.9300	C22—H22	0.9800
C4—C5	1.377 (3)	C23—O2	1.219 (2)
C4—H4	0.9300	C23—C24	1.475 (2)
C5—C6	1.375 (3)	C24—C29	1.390 (3)
C5—H5	0.9300	C24—C25	1.396 (2)
C6—N1	1.417 (2)	C25—C26	1.369 (3)
C7—N1	1.354 (2)	C25—H25	0.9300
C7—C8	1.366 (2)	C26—C27	1.383 (3)
C7—H7	0.9300	C26—H26	0.9300
C8—C9	1.424 (2)	C27—C28	1.363 (3)
C8—C16	1.507 (2)	C27—H27	0.9300
C9—N2	1.329 (2)	C28—C29	1.388 (3)
C9—C10	1.472 (3)	C28—H28	0.9300
C10—C11	1.385 (3)	C29—O1	1.357 (2)
C10—C15	1.391 (3)	C30—O1	1.435 (2)
C11—C12	1.373 (3)	C30—H30A	0.9700
C11—H11	0.9300	C30—H30B	0.9700
C12—C13	1.373 (4)	C31—C32	1.366 (2)
C12—H12	0.9300	C31—C41	1.409 (2)
C13—C14	1.366 (4)	C32—C33	1.417 (3)
C13—H13	0.9300	C32—H32	0.9300
C14—C15	1.382 (3)	C33—C34	1.361 (3)
C14—H14	0.9300	C33—H33	0.9300
C15—H15	0.9300	C34—C35	1.402 (3)
C16—C22	1.558 (2)	C34—H34	0.9300
C16—C17	1.558 (2)	C35—C41	1.403 (2)
C16—H16	0.9800	C35—C36	1.419 (3)
C17—C30	1.521 (2)	C36—C37	1.356 (3)
C17—C23	1.522 (2)	C36—H36	0.9300
C17—C18	1.602 (2)	C37—C38	1.394 (3)
C18—N3	1.465 (2)	C37—H37	0.9300
C18—C31	1.523 (2)	C38—C39	1.373 (2)
C18—C40	1.572 (2)	C38—H38	0.9300
C19—N3	1.464 (2)	C39—C41	1.394 (2)
C19—C20	1.502 (3)	C39—C40	1.467 (2)
C19—H19A	0.9700	C40—O3	1.209 (2)
C19—H19B	0.9700	N1—N2	1.355 (2)
C20—C21	1.503 (3)		
			
C2—C1—C6	119.47 (19)	C20—C21—H21B	110.7
C2—C1—H1	120.3	C22—C21—H21B	110.7
C6—C1—H1	120.3	H21A—C21—H21B	108.8
C3—C2—C1	121.0 (2)	N3—C22—C21	104.75 (14)
C3—C2—H2	119.5	N3—C22—C16	106.54 (13)
C1—C2—H2	119.5	C21—C22—C16	116.12 (15)
C4—C3—C2	119.2 (2)	N3—C22—H22	109.7
C4—C3—H3	120.4	C21—C22—H22	109.7
C2—C3—H3	120.4	C16—C22—H22	109.7
C3—C4—C5	121.1 (2)	O2—C23—C24	122.01 (16)
C3—C4—H4	119.4	O2—C23—C17	123.33 (16)
C5—C4—H4	119.4	C24—C23—C17	114.55 (14)
C6—C5—C4	119.4 (2)	C29—C24—C25	118.27 (18)
C6—C5—H5	120.3	C29—C24—C23	120.62 (16)
C4—C5—H5	120.3	C25—C24—C23	120.57 (17)
C5—C6—C1	119.88 (18)	C26—C25—C24	121.0 (2)
C5—C6—N1	119.30 (18)	C26—C25—H25	119.5
C1—C6—N1	120.81 (16)	C24—C25—H25	119.5
N1—C7—C8	108.18 (16)	C25—C26—C27	119.5 (2)
N1—C7—H7	125.9	C25—C26—H26	120.3
C8—C7—H7	125.9	C27—C26—H26	120.3
C7—C8—C9	103.82 (15)	C28—C27—C26	121.0 (2)
C7—C8—C16	126.18 (15)	C28—C27—H27	119.5
C9—C8—C16	129.38 (16)	C26—C27—H27	119.5
N2—C9—C8	111.49 (16)	C27—C28—C29	119.6 (2)
N2—C9—C10	118.38 (15)	C27—C28—H28	120.2
C8—C9—C10	130.12 (16)	C29—C28—H28	120.2
C11—C10—C15	118.20 (19)	O1—C29—C28	117.23 (17)
C11—C10—C9	121.91 (17)	O1—C29—C24	122.12 (17)
C15—C10—C9	119.88 (18)	C28—C29—C24	120.63 (18)
C12—C11—C10	121.2 (2)	O1—C30—C17	111.68 (13)
C12—C11—H11	119.4	O1—C30—H30A	109.3
C10—C11—H11	119.4	C17—C30—H30A	109.3
C11—C12—C13	119.9 (2)	O1—C30—H30B	109.3
C11—C12—H12	120.0	C17—C30—H30B	109.3
C13—C12—H12	120.0	H30A—C30—H30B	107.9
C14—C13—C12	119.8 (2)	C32—C31—C41	118.15 (15)
C14—C13—H13	120.1	C32—C31—C18	133.25 (16)
C12—C13—H13	120.1	C41—C31—C18	108.44 (14)
C13—C14—C15	120.7 (2)	C31—C32—C33	118.75 (18)
C13—C14—H14	119.6	C31—C32—H32	120.6
C15—C14—H14	119.6	C33—C32—H32	120.6
C14—C15—C10	120.1 (2)	C34—C33—C32	122.73 (19)
C14—C15—H15	120.0	C34—C33—H33	118.6
C10—C15—H15	120.0	C32—C33—H33	118.6
C8—C16—C22	110.49 (13)	C33—C34—C35	120.22 (18)
C8—C16—C17	117.93 (14)	C33—C34—H34	119.9
C22—C16—C17	105.11 (12)	C35—C34—H34	119.9
C8—C16—H16	107.6	C34—C35—C41	116.42 (18)
C22—C16—H16	107.6	C34—C35—C36	128.36 (19)
C17—C16—H16	107.6	C41—C35—C36	115.20 (19)
C30—C17—C23	105.51 (13)	C37—C36—C35	121.91 (19)
C30—C17—C16	112.68 (13)	C37—C36—H36	119.0
C23—C17—C16	113.29 (13)	C35—C36—H36	119.0
C30—C17—C18	114.16 (13)	C36—C37—C38	121.9 (2)
C23—C17—C18	107.67 (12)	C36—C37—H37	119.1
C16—C17—C18	103.64 (12)	C38—C37—H37	119.1
N3—C18—C31	111.70 (13)	C39—C38—C37	118.2 (2)
N3—C18—C40	103.76 (12)	C39—C38—H38	120.9
C31—C18—C40	102.05 (13)	C37—C38—H38	120.9
N3—C18—C17	106.68 (12)	C38—C39—C41	120.36 (17)
C31—C18—C17	120.46 (13)	C38—C39—C40	132.08 (18)
C40—C18—C17	110.98 (12)	C41—C39—C40	107.56 (15)
N3—C19—C20	101.60 (16)	O3—C40—C39	127.25 (16)
N3—C19—H19A	111.5	O3—C40—C18	124.79 (15)
C20—C19—H19A	111.5	C39—C40—C18	107.92 (14)
N3—C19—H19B	111.5	C39—C41—C35	122.50 (17)
C20—C19—H19B	111.5	C39—C41—C31	113.72 (15)
H19A—C19—H19B	109.3	C35—C41—C31	123.71 (17)
C19—C20—C21	105.65 (18)	C7—N1—N2	111.42 (14)
C19—C20—H20A	110.6	C7—N1—C6	128.37 (16)
C21—C20—H20A	110.6	N2—N1—C6	120.09 (14)
C19—C20—H20B	110.6	C9—N2—N1	105.09 (14)
C21—C20—H20B	110.6	C19—N3—C18	119.16 (13)
H20A—C20—H20B	108.7	C19—N3—C22	106.39 (14)
C20—C21—C22	105.13 (16)	C18—N3—C22	106.33 (12)
C20—C21—H21A	110.7	C29—O1—C30	114.24 (13)
C22—C21—H21A	110.7		
			
C6—C1—C2—C3	0.4 (4)	C23—C24—C29—O1	6.7 (3)
C1—C2—C3—C4	−0.3 (4)	C25—C24—C29—C28	−0.1 (3)
C2—C3—C4—C5	0.1 (4)	C23—C24—C29—C28	−171.65 (16)
C3—C4—C5—C6	−0.1 (4)	C23—C17—C30—O1	63.56 (16)
C4—C5—C6—C1	0.2 (4)	C16—C17—C30—O1	−172.34 (13)
C4—C5—C6—N1	−178.9 (2)	C18—C17—C30—O1	−54.46 (18)
C2—C1—C6—C5	−0.4 (3)	N3—C18—C31—C32	−68.8 (2)
C2—C1—C6—N1	178.77 (19)	C40—C18—C31—C32	−179.13 (18)
N1—C7—C8—C9	0.1 (2)	C17—C18—C31—C32	57.5 (3)
N1—C7—C8—C16	171.79 (16)	N3—C18—C31—C41	106.35 (15)
C7—C8—C9—N2	0.2 (2)	C40—C18—C31—C41	−3.94 (16)
C16—C8—C9—N2	−171.10 (17)	C17—C18—C31—C41	−127.31 (15)
C7—C8—C9—C10	178.72 (18)	C41—C31—C32—C33	1.2 (3)
C16—C8—C9—C10	7.4 (3)	C18—C31—C32—C33	175.98 (17)
N2—C9—C10—C11	−140.66 (19)	C31—C32—C33—C34	−1.2 (3)
C8—C9—C10—C11	40.9 (3)	C32—C33—C34—C35	0.1 (3)
N2—C9—C10—C15	38.5 (2)	C33—C34—C35—C41	1.0 (3)
C8—C9—C10—C15	−140.0 (2)	C33—C34—C35—C36	−177.2 (2)
C15—C10—C11—C12	1.0 (3)	C34—C35—C36—C37	178.0 (2)
C9—C10—C11—C12	−179.88 (19)	C41—C35—C36—C37	−0.2 (3)
C10—C11—C12—C13	0.1 (3)	C35—C36—C37—C38	−0.3 (4)
C11—C12—C13—C14	−0.9 (4)	C36—C37—C38—C39	0.6 (3)
C12—C13—C14—C15	0.6 (4)	C37—C38—C39—C41	−0.3 (3)
C13—C14—C15—C10	0.4 (3)	C37—C38—C39—C40	179.67 (19)
C11—C10—C15—C14	−1.2 (3)	C38—C39—C40—O3	−3.1 (3)
C9—C10—C15—C14	179.65 (18)	C41—C39—C40—O3	176.84 (17)
C7—C8—C16—C22	−34.1 (2)	C38—C39—C40—C18	174.97 (19)
C9—C8—C16—C22	135.43 (18)	C41—C39—C40—C18	−5.06 (18)
C7—C8—C16—C17	86.8 (2)	N3—C18—C40—O3	67.4 (2)
C9—C8—C16—C17	−103.7 (2)	C31—C18—C40—O3	−176.41 (17)
C8—C16—C17—C30	−5.0 (2)	C17—C18—C40—O3	−46.9 (2)
C22—C16—C17—C30	118.65 (15)	N3—C18—C40—C39	−110.78 (14)
C8—C16—C17—C23	114.74 (16)	C31—C18—C40—C39	5.43 (16)
C22—C16—C17—C23	−121.65 (14)	C17—C18—C40—C39	134.99 (14)
C8—C16—C17—C18	−128.87 (14)	C38—C39—C41—C35	−0.2 (3)
C22—C16—C17—C18	−5.26 (15)	C40—C39—C41—C35	179.83 (16)
C30—C17—C18—N3	−138.42 (14)	C38—C39—C41—C31	−177.43 (16)
C23—C17—C18—N3	104.79 (14)	C40—C39—C41—C31	2.6 (2)
C16—C17—C18—N3	−15.49 (15)	C34—C35—C41—C39	−177.94 (17)
C30—C17—C18—C31	92.96 (18)	C36—C35—C41—C39	0.4 (3)
C23—C17—C18—C31	−23.82 (19)	C34—C35—C41—C31	−1.0 (3)
C16—C17—C18—C31	−144.10 (15)	C36—C35—C41—C31	177.37 (17)
C30—C17—C18—C40	−26.04 (18)	C32—C31—C41—C39	177.11 (15)
C23—C17—C18—C40	−142.82 (14)	C18—C31—C41—C39	1.1 (2)
C16—C17—C18—C40	96.90 (14)	C32—C31—C41—C35	−0.1 (3)
N3—C19—C20—C21	−35.3 (3)	C18—C31—C41—C35	−176.11 (15)
C19—C20—C21—C22	17.0 (3)	C8—C7—N1—N2	−0.4 (2)
C20—C21—C22—N3	7.8 (2)	C8—C7—N1—C6	−176.41 (17)
C20—C21—C22—C16	−109.3 (2)	C5—C6—N1—C7	168.8 (2)
C8—C16—C22—N3	152.70 (14)	C1—C6—N1—C7	−10.3 (3)
C17—C16—C22—N3	24.48 (17)	C5—C6—N1—N2	−6.9 (3)
C8—C16—C22—C21	−91.11 (18)	C1—C6—N1—N2	173.96 (17)
C17—C16—C22—C21	140.67 (15)	C8—C9—N2—N1	−0.4 (2)
C30—C17—C23—O2	144.65 (17)	C10—C9—N2—N1	−179.14 (15)
C16—C17—C23—O2	20.9 (2)	C7—N1—N2—C9	0.5 (2)
C18—C17—C23—O2	−93.06 (19)	C6—N1—N2—C9	176.88 (16)
C30—C17—C23—C24	−39.16 (18)	C20—C19—N3—C18	161.15 (17)
C16—C17—C23—C24	−162.88 (14)	C20—C19—N3—C22	41.2 (2)
C18—C17—C23—C24	83.13 (16)	C31—C18—N3—C19	45.1 (2)
O2—C23—C24—C29	−176.82 (17)	C40—C18—N3—C19	154.27 (15)
C17—C23—C24—C29	6.9 (2)	C17—C18—N3—C19	−88.46 (17)
O2—C23—C24—C25	11.8 (3)	C31—C18—N3—C22	165.10 (13)
C17—C23—C24—C25	−164.43 (16)	C40—C18—N3—C22	−85.70 (14)
C29—C24—C25—C26	−0.7 (3)	C17—C18—N3—C22	31.56 (16)
C23—C24—C25—C26	170.91 (19)	C21—C22—N3—C19	−30.85 (19)
C24—C25—C26—C27	0.8 (3)	C16—C22—N3—C19	92.72 (16)
C25—C26—C27—C28	−0.3 (4)	C21—C22—N3—C18	−158.84 (14)
C26—C27—C28—C29	−0.5 (3)	C16—C22—N3—C18	−35.27 (16)
C27—C28—C29—O1	−177.77 (18)	C28—C29—O1—C30	−164.59 (15)
C27—C28—C29—C24	0.6 (3)	C24—C29—O1—C30	17.0 (2)
C25—C24—C29—O1	178.25 (16)	C17—C30—O1—C29	−53.90 (18)

### Hydrogen-bond geometry (Å, º)

**Table:**

*D*—H···*A*	*D*—H	H···*A*	*D*···*A*	*D*—H···*A*
C22—H22···O3^i^	0.98	2.47	3.273 (2)	138

Symmetry code: (i) −*x*, −*y*+1, −*z*+1.
